# Prompt clinical and electrophysiological remission of refractory peripheral neuropathy in long-standing rheumatoid arthritis with tocilizumab: a case report

**DOI:** 10.3389/fimmu.2026.1770170

**Published:** 2026-02-12

**Authors:** Hai Huang, Xuexian Zhong, Shan Kuang, Jinhui Tan, Linghua Tan, Bo Li

**Affiliations:** 1 Department of Health Management, People’s Hospital of Longhua, Shenzhen, Guangdong, China; 2 Yikangyuan Community Health Center, Bao’an Center Hospital of Shenzhen, Shenzhen, Guangdong, China; 3 Department of emergency and critical care medicine, Shenzhen Fuyong People’s Hospital, Shenzhen, Guangdong, China; 4 Department of Rheumatology and Immunology, People’s Hospital of Longhua, Shenzhen, Guangdong, China; 5 Department of Health Management, Jiangmen Wuyi Hospital of Chinese Medicine, Jiangmen, Guangdong, China

**Keywords:** arthritis, rheumatoid, glucocorticoids, peripheral nervous system diseases, tocilizumab, vasculitis

## Abstract

Peripheral neuropathy (PN) is a debilitating extra-articular manifestation of rheumatoid arthritis (RA), frequently driven by chronic inflammation and vasculitis. Its management remains challenging owing to limited evidence and the adverse effects associated with long-term glucocorticoids (GCs) use. We present a 48−year−old woman with 21-year seropositive RA who developed severe, progressive PN manifesting as multifocal asymmetric sensorimotor deficits. Electrophysiology confirmed axonal damage, and cerebrospinal fluid (CSF) showed albuminocytologic dissociation. Previous therapies, including methotrexate, etanercept, and tofacitinib, failed to control neuropathy. During a severe flare with high inflammatory markers, she received intravenous methylprednisolone followed by the interleukin-6 (IL-6) receptor antagonist tocilizumab. Within days, neuropathic symptoms and arthritis improved. After 12 months of monthly tocilizumab, inflammatory markers normalized, and repeat nerve−conduction studies demonstrated significant recovery. This case highlights that tocilizumab, by targeting the IL-6 pathway pivotal to RA-associated vasculitis and systemic inflammation, can induce rapid remission of refractory PN while facilitating glucocorticoid tapering. It supports the use of biologic agents with distinct mechanisms in managing complex extra−articular RA.

## Introduction

Rheumatoid arthritis (RA) is a chronic, systemic autoimmune disorder characterized by persistent synovial inflammation, leading to progressive joint destruction, functional disability, and increased mortality (1–3). A critical aspect of RA is its association with various extra-articular manifestations (EAMs) that significantly contribute to disease morbidity and complexity (4). Among these, peripheral neuropathy (PN) represents one of the most common and debilitating complications, with a reported prevalence ranging widely from 10% to over 75% in different cohorts, largely due to variations in diagnostic criteria and study populations (5–7). This heterogeneity underscores the diagnostic challenges, as a substantial proportion of PN cases are subclinical, detectable only through systematic electrophysiological studies (6, 7). The clinical spectrum of RA-associated PN is diverse, encompassing distal symmetric sensory or sensorimotor polyneuropathy (the most frequent form), mononeuritis multiplex, and entrapment neuropathies (4, 6, 8, 9). Key risk factors for developing PN encompass several domains: longer disease duration; high disease activity, evidenced by elevated inflammatory markers and a high Disease Activity Score in 28 joints (DAS-28); seropositivity for rheumatoid factor (RF) and anti-cyclic citrullinated peptide antibody (ACPA); and the presence of other systemic features, including rheumatoid nodules or vasculitis (4, 6–8, 10).

The pathogenesis of PN in RA is multifactorial, driven primarily by chronic systemic inflammation (11). Pro-inflammatory cytokines, including tumor necrosis factor-alpha (TNF-α) and interleukin-6 (IL-6), contribute directly to neural dysfunction and hyperalgesia (11). A pivotal mechanism is necrotizing vasculitis of the vasa nervorum, leading to ischemic nerve injury, which often manifests as acute-onset mononeuritis multiplex or asymmetric polyneuropathy with axonal loss on electrophysiology (11). The management of RA-associated PN remains a significant therapeutic challenge. This is exacerbated by the scarcity of randomized controlled trials specifically addressing this complication (11). Although high-dose glucocorticoids (GCs) are the cornerstone of initial therapy for severe, vasculitic PN due to their rapid immunosuppressive effects (11, 12), their long-term use is limited by a well-documented profile of dose-dependent adverse effects, including osteoporosis, diabetes, cardiovascular events, and increased infection risk, necessitating a swift transition to steroid-sparing agents (11, 13).

Conventional synthetic disease-modifying antirheumatic drugs (csDMARDs) like methotrexate often provide inadequate control for progressive PN (8, 14, 15). Biologic DMARDs (bDMARDs) offer alternative pathways. While TNF-α inhibitors are effective for arthritis, their association with paradoxical induction or exacerbation of demyelinating neuropathies in some patients limits their utility in this context (16). IL-6 is a key cytokine in RA pathogenesis, driving systemic inflammation, acute-phase responses, and endothelial activation implicated in vasculitis (17). Tocilizumab, a monoclonal antibody targeting the IL-6 receptor, has demonstrated efficacy in controlling refractory articular disease and shows promise in managing systemic manifestations, including vasculitis, by comprehensively suppressing the inflammatory milieu (17, 18). Its potent steroid-sparing effect makes it a compelling choice for managing complex EAMs like PN (18). This case report describes the successful management of a patient with severe, refractory RA-associated PN using tocilizumab, highlighting the importance of targeted therapy in achieving remission while minimizing glucocorticoid toxicity (18).

## Case presentation

### Patient information and chief complaints

**Figure 1:**
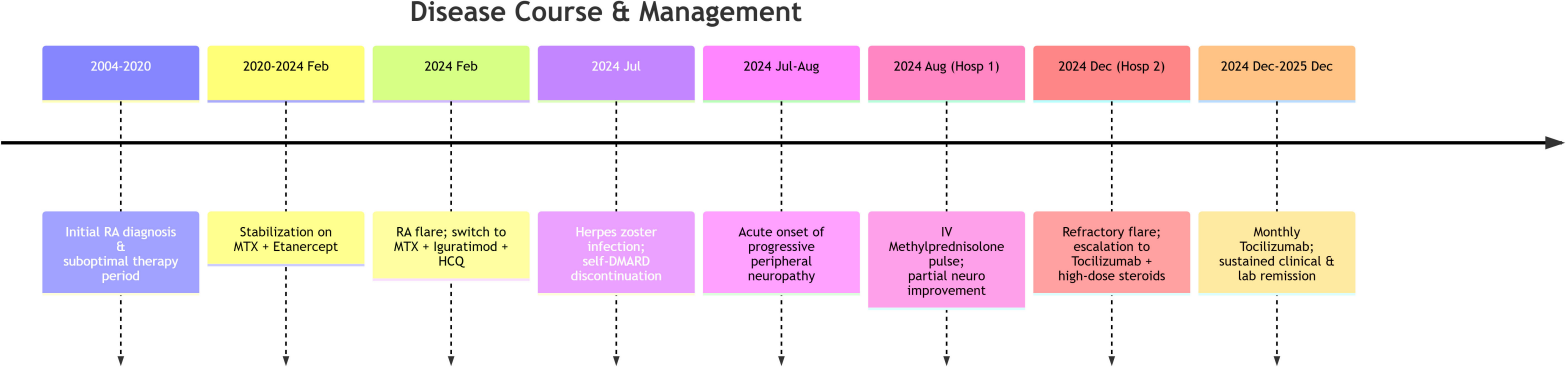
Timeline of disease progression and therapeutic interventions in a patient with rheumatoid arthritis (RA)-associated refractory peripheral neuropathy (PN). RA, rheumatoid arthritis; MTX, methotrexate; HCQ, hydroxychloroquine; DMARD, disease-modifying antirheumatic drug; IV, intravenous; Hosp, hospitalization.

A 48-year-old female presented to our Rheumatology department with a 21-year history of polyarticular pain and swelling, and a 1-year history of recurrent bilateral lower limb numbness and weakness. The patient’s primary concerns at the most recent admission were worsening neuropathic symptoms and active articular disease, signifying a flare of her underlying condition. The complex chronology of her disease evolution, key interventions, and outcomes is summarized in **Figure 1**.

### History of present illness and evolution of RA

The patient’s autoimmune journey began in 2004 (age 27) with the insidious onset of symmetrical polyarthritis involving the proximal interphalangeal joints, metacarpophalangeal joints, wrists, shoulders, knees, and feet, accompanied by morning stiffness lasting over one hour. A diagnosis of RA was established at a local hospital. Initial treatment with methotrexate (MTX) was effective but was self-discontinued due to side effect concerns. Subsequently, disease control was suboptimal, involving periods of traditional Chinese medicine (3 years, ineffective), etanercept (approximately 2 years, discontinued due to cost), and tofacitinib (1 year, ineffective).

In 2020, a more structured regimen was initiated, consisting of subcutaneous etanercept (50 mg weekly) combined with oral MTX (12.5 mg weekly). This provided a period of relative stability until February 2024, when she experienced a flare with swelling and pain in her metacarpophalangeal joints. Etanercept was stopped, and her regimen was adjusted to MTX (increased to 15 mg weekly), iguratimod (25 mg twice daily), and hydroxychloroquine (200 mg daily).

A pivotal clinical turning point occurred in July 2024. The patient developed herpes zoster in the right thoracic region. During this acute infection, she self-discontinued all DMARDs. Shortly after the resolution of the shingles rash, she rapidly developed new neurological symptoms: numbness and hypoesthesia localized to the right knee and lower leg, followed by left foot numbness. Notably, she experienced significant right foot dorsiflexion weakness. This prompted her first hospitalization in August 2024.

### Past medical history and key hospitalizations

The patient’s history is significant for multiple RA flares and the development of irreversible joint damage. Crucially, she had no documented history of diabetes, hypothyroidism, or other conditions commonly associated with PN. There was no relevant family history of hereditary neuropathies, diabetes mellitus, or autoimmune diseases.

Hospitalization 1 (August 2024): Admitted for acute-onset PN. Key findings included elevated inflammatory markers (erythrocyte sedimentation rate (ESR) 40 mm/h, high-sensitivity C-reactive protein (hs-CRP) 26.1 mg/L) and high titers of RA-associated antibodies (RF 187.7 IU/mL, ACPA 58.5 U/mL) (**Table 1**). CSF analysis showed mildly elevated protein (36.7 mg/dL) with normal cell count. The nerve conduction studies (NCS) and electromyography (EMG) conducted on August 5, 2024, have confirmed a moderate-to-severe lesion of the right common peroneal nerve (**Table 2**). A diagnosis of RA-associated PN was made. She received intravenous methylprednisolone therapy (80 mg daily for 5 days), leading to mild symptomatic improvement. Follow-up NCS/EMG on August 16 showed persistent but improved multifocal peripheral nerve involvement in the lower limbs (**Table 2**). She was discharged on a regimen of iguratimod, hydroxychloroquine, and neuropathic pain agents (pregabalin, oxcarbazepine).

**Table 1:** Serial changes in inflammatory and serologic markers before and after treatment escalation.

Index	2024-08-06	2024-12-02	2025-04-03	2025-08-06	2025-12-05
ESR (mm/h)	40	56.1	3.96	4.64	4.34
hs-CRP (mg/L)	26.1	45.8	0.6	4.83	7.05
RF (IU/mL)	187.7	496.5	358.9	321.3	300.5
ACPA (U/mL)	58.5	53.2	21.1	13.6	18.1

The reference ranges for ESR, hs-CRP, RF, and ACPA are 0–15 mm/h, 0–10 mg/L, 0–14 IU/mL, and 0–5 U/mL, respectively. ESR, erythrocyte sedimentation rate; hs-CRP, high-sensitivity C-reactive protein; RF, rheumatoid factor; ACPA, anti-cyclic citrullinated peptide antibody.

**Table 2:** Sequential electromyography and nerve conduction study findings throughout the clinical course.

Date	Nerve studied & key quantitative parameters (distal CMAP amplitude; sensory amplitude)	Clinical context & interpretation
2024-08-05	R. Peroneal (Motor to EDB): Ankle: 0.1 mV (NR >2.5); Fibular Head: 0.1 mV. L. Tibial (Motor to AH): Ankle: 7.1 mV (NR >3.0). Sural N. (Sensory): R: 7.8 µV (NR >5.0); L: 6.6 µV.	Post-herpes zoster, acute neuropathic onset. Findings indicate an asymmetric, axonal-pattern, multifocal neuropathy (right peroneal severe, left tibial moderate).
2024-08-16	R. Peroneal (Motor to EDB): Ankle: 0.1 mV; CV: 36.4 m/s (NR >38). L. Tibial (Motor to AH): Ankle: 5.3 mV. Sural N. (Sensory): Amplitudes severely reduced (1.0-1.6 µV).	10 days post-IV methylprednisolone (80 mg/day). Confirmed bilateral, multifocal sensorimotor involvement. Mild symptomatic improvement but persistent significant axonal loss on NCS.
2024-12-04	R. Peroneal (Motor to EDB): Ankle: NR (0 mV); Fibular Head: 0.1 mV. L. Tibial (Motor to AH): Ankle: 5.4 mV. R. Tibial (Motor to AH): Ankle: 2.6 mV. Sural N. (Sensory): 3.7 µV (Bilat.).	Major systemic & neurological flare prior to therapy escalation. Demonstrates clear progression: worsened right peroneal, new right tibial, and worsened left tibial involvement.
2024-12-16	R. Peroneal (Motor to TA): Ankle: NR; Fibular Head: 1.0 mV. L. Tibial (Motor to AH): Ankle: 5.3 mV. R. Tibial (Motor to AH): Ankle: 2.4 mV. Sural N. (Sensory): 2.5-4.3 µV.	~2 weeks after first dose of tocilizumab (Dec 5). Objective early improvement: bilateral tibial CMAPs stable or slightly improved compared to Dec-4, suggesting halted progression.
2025-12-05	R. Peroneal (Motor to EDB): Ankle: 0.1 mV; CV: 28.0 m/s (↓). L. Peroneal (Sensory): Amplitude: 1.5 µV (↓). Bilateral Tibial (Motor): CMAPs normalized (6.1-8.9 mV). R. Sural N.: NR.	12 months of monthly tocilizumab maintenance. Definite objective recovery: Near-complete resolution of tibial nerve motor deficits. Residual severe right peroneal damage.

AH, abductor hallucis; CMAP, compound muscle action potential; CV, conduction velocity; EDB, extensor digitorum brevis; L, left; NR, normal reference range; R, right; TA, tibialis anterior; ↓, reduced compared to normal lower limit.

Hospitalization 2 (December 2024): Represented with a severe systemic and worsening neurological symptoms. In the absence of any intercurrent infection, she reported intensified bilateral lower limb numbness and pain, cervical pain, and active polyarthritis with morning stiffness. Inflammatory markers were markedly elevated (ESR 56.1 mm/h, hs-CRP 45.8 mg/L, RF 496.5 IU/mL) (**Table 1**). Repeat NCS/EMG on December 4, 2024, demonstrated significant worsening and extension of the polyneuropathy, revealing severe right common peroneal nerve, moderate-to-severe left tibial nerve, and mild-to-moderate right tibial nerve involvement, affecting both motor and sensory fibers (**Table 2**). This objective evidence of progressive nerve injury despite previous therapy defined her condition as refractory.

### Physical examination findings (at second admission, December 2024)

Musculoskeletal System: Swan-neck deformities of the fingers and foot deformities consistent with long-standing RA. Active synovitis with swelling and tenderness was noted in the metacarpophalangeal joints.

Neurological System: Muscle tone was normal globally. Muscle strength was graded as 5/5 in all limbs, indicating substantial recovery from the severe right foot drop (0/5) documented in August. A distinct sensory disturbance was present: pinprick hypersensitivity was observed bilaterally below the mid-tibial level. Vibration sense (tested with a 128-Hz tuning fork) was diminished at both medial malleoli. Light touch sensation was decreased in a stocking distribution up to the mid-shins. Proprioception at the great toes was intact. Temperature sensation was normal. Deep tendon reflexes were symmetrically diminished in all four limbs. Plantar responses were flexor. No meningeal signs were present.

### Diagnostic investigations

A comprehensive workup was performed to exclude alternative causes of neuropathy and assess disease activity.

1. Serological studies: Consistently showed high-titer seropositivity for RF and ACPA. Antinuclear antibody (ANA) and anti-U1-snRNP antibody were positive. Antibodies associated with vasculitis (ANCA), paraneoplastic syndromes, and specific peripheral nerve targets were negative. Serial monitoring of ESR and hs-CRP provided clear correlation with clinical disease activity.
2. All neurophysiological studies (NCS/EMG) were performed and interpreted by board-certified neurologists/neurophysiologists. CSF analysis (August 2024): Revealed albuminocytologic dissociation: protein 36.7 mg/dL (reference <45 mg/dL) with a normal white cell count (3.3/µL; total nucleated cell count 3.3/µL).
3. Neurophysiological studies (serial NCS/EMG): Provided the cornerstone objective evidence. The initial study (Aug 5, 2024) revealed severe axonal injury of the right common peroneal nerve (distal compound muscle action potential (CMAP) amplitude: 0.1 mV, normal >2.5 mV) and moderate injury of the left tibial nerve. A follow-up study on Aug 16 showed persistent bilateral multifocal involvement, with severely reduced sensory amplitudes (e.g., sural nerve sensory nerve action potentials (SNAPs): 1.0-1.6 μV, normal >5.0 μV). By Dec 4, 2024, during a major flare, studies confirmed significant worsening: the right peroneal motor response was unrecordable at the ankle, left tibial CMAP was 5.4 mV, and right tibial CMAP was 2.6 mV, with universally low sensory amplitudes (bilateral sural: 3.7 μV). This evolution from a focal neuropathy to an asymmetric, multifocal, axonal sensorimotor polyneuropathy is detailed in **Table 2**. Notably, upper limb NCS (median and ulnar nerves) performed in all five sessions were consistently normal, confirming the neuropathy was predominantly distributed in the lower limbs.
4. Imaging: MRI of the thoracic-lumbar spine and cervical spine showed only degenerative changes without evidence of cord compression, radiculopathy, or leptomeningeal enhancement, effectively ruling out structural causes for her symptoms.

Diagnosis: Based on the chronic, deforming polyarthritis, seropositivity, and the exclusion of other causes, the primary diagnosis was refractory seropositive RA. The secondary diagnosis of RA-associated PN was due to an acute-onset, stepwise progressive nerve injury that was confirmed by NCS/EMG. This injury was temporally linked to RA flares and responded positively to immunosuppression, suggesting that it was likely mediated by autoimmune vasculitis.

### Diagnostic reasoning and exclusion of alternatives

The diagnosis of RA-associated vasculitic neuropathy was favored based on: (1) temporal correlation with RA disease activity, (2) seropositivity and high inflammatory markers, (3) an asymmetric, multifocal, axonal sensorimotor pattern on serial NCS/EMG, and (4) response to immunosuppression. We actively excluded alternatives: Chronic inflammatory demyelinating polyneuropathy (CIDP)/Lewis-Sumner syndrome (MADSAM) was unlikely due to the absence of demyelinating features (e.g., conduction blocks, severe conduction velocity slowing) across all five studies and the acute, stepwise progression. Post-herpetic radiculopathy was considered, but the neuropathy rapidly evolved beyond the affected dermatome into a multifocal pattern. Guillain-Barré syndrome variants were excluded due to the chronic, relapsing course over months. Drug-induced neuropathy was excluded as no known neurotoxic agents were used at onset. Metabolic causes (diabetes, B12 deficiency, hypothyroidism) were ruled out by laboratory testing.

### Therapeutic intervention and clinical course

Given the refractory nature of both arthritis and neuropathy to previous DMARDs and TNF-inhibition, therapy was escalated during the second hospitalization.

1. Induction therapy: Intravenous methylprednisolone at a dose of 80 mg daily (approximately 1 mg/kg/day) was administered for 7 days. On December 5, 2024, tocilizumab (400 mg IV) was initiated. Oral prednisone was started at 50 mg/day and tapered over the following months. Neuropathic pain medications (pregabalin, oxcarbazepine) were gradually withdrawn within 3 months of symptomatic improvement. Tocilizumab was maintained at monthly intervals (every 4 weeks).
2. Clinical response: The combination therapy yielded a rapid and positive response. Joint pain, morning stiffness, and neuropathic pain began to subside within days.
3. Discharge and maintenance regimen: The patient was discharged on a tapering course of oral prednisone (starting at 50 mg daily) and continued monthly intravenous tocilizumab infusions. Hydroxychloroquine was maintained.
4. Follow-up and outcomes: Adherence to monthly tocilizumab resulted in sustained remission. At the most recent follow-up in December 2025, the patient reported resolution of left lower limb numbness, with only residual numbness in the anterior right foot. Arthralgia was absent. Inflammatory markers had normalized (ESR 4.34 mm/h, hs-CRP 0.34 mg/L) (**Table 1**). Follow-up NCS/EMG after 12 months of tocilizumab therapy (December 5, 2025) documented objective, progressive improvement. Motor amplitudes of the bilateral tibial nerves had normalized (left: 8.9 mV, right: 6.1 mV). The right common peroneal nerve, while still severely damaged, showed a recordable, albeit very low, distal CMAP (0.1 mV). Sensory amplitudes, however, showed a more modest recovery, with the left sural nerve at 1.5 μV and the right remaining unrecordable. These findings correlate with the patient’s clinical recovery and are detailed in **Table 2**.

### Patient perspective

The patient expressed profound relief at the stabilization of her condition following years of debilitating and progressive symptoms. She reported a significant improvement in quality of life and the ability to perform daily activities without pain. Informed consent was obtained for the publication of this case report.

## Discussion

The present case of a patient with long-standing, seropositive RA who developed severe, progressive PN and achieved rapid remission following tocilizumab therapy, illuminates critical aspects in the management of this debilitating extra-articular manifestation. RA-associated PN represents a complex interplay of chronic inflammation (5), vasculitic injury (8), and iatrogenic factors, demanding a therapeutic strategy that balances efficacy with long-term safety (19). In this discussion, we contextualize our therapeutic strategy by analyzing: 1) the epidemiologic and pathogenic links between RA and PN; 2) the established role and significant limitations of high-dose GCs; 3) the compelling rationale for selecting IL-6 receptor blockade with tocilizumab in this scenario; and 4) the integrative treatment approach that led to a successful outcome.

### Epidemiology, clinical spectrum, and pathogenesis of RA-associated PN

Peripheral neuropathy (PN) is a common yet underdiagnosed extra-articular feature of RA, with reported prevalence ranging widely from 10% to over 75%, a variance attributable to differences in diagnostic methods and study populations (4, 5). A significant proportion of cases are subclinical, detectable only through electrophysiological studies (6). The clinical presentations are heterogeneous, encompassing distal symmetric sensory or sensorimotor polyneuropathy (the most common form), mononeuritis multiplex, and entrapment neuropathies like carpal tunnel syndrome (6, 19). Key risk factors for developing PN include longer disease duration, high disease activity (elevated ESR, CRP, DAS-28), seropositivity for RF and ACPA, and the presence of other systemic features such as rheumatoid nodules or vasculitis (6, 8). Our patient’s profile—with a 21-year history of active, seropositive RA, high inflammatory markers, and functional disability—placed her in a high-risk category for severe neuropathic complications.

The pathogenesis of PN in RA is multifactorial. Chronic systemic inflammation being a central driver (10), and pro-inflammatory cytokines directly contributing to neural dysfunction and hyperalgesia (20). A primary and severe mechanism is necrotizing vasculitis of the vasa nervorum (8), leading to ischemic nerve injury, which typically manifests as acute-onset mononeuritis multiplex (8, 9, 21) or asymmetric polyneuropathy with predominant axonal loss on electrophysiology, as seen in our patient. The presence of albuminocytologic dissociation in the CSF of our patient further supports an inflammatory, immune-mediated radiculoneuropathy. Beyond active vasculitis, chronic synovitis can cause compressive neuropathies (6), while longstanding disease and metabolic factors may lead to a distal symmetric axonal neuropathy (6, 7). Importantly, certain DMARDs have also been implicated in inducing or exacerbating PN (14, 22, 23), adding a layer of diagnostic and therapeutic complexity.

### The dual-edged sword: high-dose GCs in RA-associated PN

High-dose GCs remain a first-line intervention for severe, life- or organ-threatening extra-articular manifestations of RA, including active vasculitis and rapidly progressive PN (11). Their rapid, potent anti-inflammatory and immunosuppressive effects can halt acute immune-mediated nerve damage. In our case, intravenous methylprednisolone therapy was initiated to urgently suppress the presumed vasculitic process driving the stepwise neurological decline.

However, the profound and dose-dependent adverse effects of GCs render their long-term use highly problematic, a concern extensively documented in the provided literature (13, 24). The toxicity profile is broad, encompassing cardiovascular events (hypertension, accelerated atherosclerosis) (13, 24), metabolic disturbances (diabetes mellitus, dyslipidemia) (13, 24), osteoporosis with increased fracture risk (13, 24), osteonecrosis, cataracts, and a significantly elevated risk of serious infections (13, 24). The risk of infections, including opportunistic and reactivation of latent tuberculosis or herpes zoster, is of particular concern in RA patients already on other immunosuppressants (13). Our patient’s history of recent herpes zoster infection prior to presentation highlights this vulnerability.

Expert guidelines strongly advocate for using the lowest effective GCs dose for the shortest possible duration, employing them primarily as a “bridge” to control acute inflammation until a steroid-sparing immunosuppressive agent takes full effect (12, 13, 24). Prolonged use, especially at doses greater than 7.5–10 mg/day of prednisone equivalent, is associated with accrual of irreversible organ damage and increased mortality (12, 13, 24). Therefore, while our initial use of GCs was justified, a definitive, GCs-sparing strategy was imperative. The failure of the patient’s neuropathy to improve significantly on moderate-dose oral prednisone alone underscored the need for a more targeted and potent immunosuppressive agent.

### Rationale for selecting tocilizumab: targeting the IL-6 pathway

The selection of tocilizumab as the foundational therapy for this case was underpinned by a strong pathophysiological and clinical rationale, informed by the understanding of IL-6’s central role in RA and its systemic complications.

Pathophysiological basis: IL-6 is a pleiotropic cytokine critically involved in the pathogenesis of RA (25, 26). It drives acute-phase responses (e.g., production of CRP and ESR elevation) (25, 27–29), B-cell differentiation into autoantibody-producing plasma cells (25, 26), T-cell activation (25, 26), and osteoclastogenesis, contributing to both joint destruction and systemic inflammation (25, 26). Crucially, IL-6 is a key mediator of endothelial activation and vascular inflammation (26, 30–32), directly implicating it in the development of vasculitic processes that can damage peripheral nerves. In our patient, the presence of severe systemic inflammation, as evidenced by markedly elevated acute-phase reactants (ESR and CRP) in the context of active polyarthritis and progressive neuropathy, is pathophysiologically linked to the overproduction of pro-inflammatory cytokines, notably IL-6 (7, 9, 33). Furthermore, IL-6 is a pivotal mediator of endothelial activation and vascular inflammation (32). It promotes the expression of adhesion molecules, facilitates leukocyte recruitment (30), and contributes to the breakdown of the blood-nerve barrier. In the context of RA, this endothelial dysfunction can culminate in necrotizing vasculitis of the vasa nervorum, leading to ischemic axonal injury (9) – the electrophysiological hallmark of the severe, asymmetric neuropathy observed in our patient. The rapid normalization of acute-phase reactants (ESR, CRP) following tocilizumab initiation not only reflects control of systemic inflammation but also suggests suppression of this IL-6-driven vascular inflammatory process (32), thereby creating a permissive environment for nerve recovery.

In this clinical context, our therapeutic sequence was deliberate. The initial use of intravenous methylprednisolone at 80 mg daily (approximately 1 mg/kg/day, rather than a standard high-dose pulse) served as a critical “bridge” therapy. This intermediate dose aimed to rapidly dampen the severe vasculitic inflammation while minimizing the risk of infection in a patient with recent herpes zoster, providing a window for the definitive steroid-sparing agent to take effect. The subsequent introduction of tocilizumab was therefore strategic, targeting the IL-6 pathway identified as central to her disease pathology. Given the well-established central role of IL-6 in driving synovitis, acute-phase responses, and vascular inflammation in RA, the blockade of the IL-6 receptor with tocilizumab represented a rational and targeted strategy to address the underlying inflammatory drive of both her articular and neuropathic disease (33).

Clinical efficacy in refractory RA and systemic manifestations: Tocilizumab has demonstrated superior efficacy in controlling articular inflammation in RA, including in patients with inadequate response to anti-TNF agents (17, 25, 34). Beyond joints, emerging evidence and case reports suggest its potential benefit in managing various extra-articular manifestations, including vasculitis and systemic inflammation (18, 20). While large randomized trials specifically for PN are lacking, the drug’s ability to comprehensively suppress the inflammatory milieu that fuels vasculitis and neural damage provides a strong therapeutic premise (18, 20, 25). Documented cases describe improvement in RA-related neuropathies and vasculitic skin ulcers following tocilizumab treatment (18, 20).

Advantages over alternative biologics: The patient had previously failed an anti-TNF agent (etanercept). Anti-TNF agents, while effective for arthritis, have a recognized paradoxical effect of potentially inducing or exacerbating demyelinating neuropathies and other autoimmune phenomena in a subset of patients (16). Switching to a biologic with a different mechanism of action was therefore indicated (34). Rituximab (a B-cell depletor) is another effective option for vasculitis (35), but tocilizumab was chosen for its rapid onset of action, its excellent effect on systemic inflammation (as reflected by acute-phase reactants), and its favorable GCs-sparing effect, which aligned with the urgent need to control the disease and taper prednisone quickly (17, 25).

### Integration of therapies and achievement of treatment goals

The successful outcome in this case was achieved not by a single drug but through an integrated, sequential treatment strategy (11). The initial high-dose GCs provided immediate immunosuppression to arrest the active vasculitic nerve injury (9–11). Tocilizumab was introduced concurrently as the cornerstone maintenance therapy to provide deep and sustained IL-6 pathway inhibition (11, 17, 25, 34). The subsequent rapid and consistent normalization of ESR and CRP served as an objective biomarker confirming effective control of the systemic inflammatory drive (11, 17, 25, 34).

The clinical correlation was striking: the resolution of arthralgia and the dramatic improvement in neuropathic symptoms (numbness, weakness, and pain) paralleled the biochemical response. The 12-month follow-up nerve conduction study, showing significant improvement, provided objective electrophysiological evidence of nerve repair and functional recovery once the inflammatory insult was removed. This allowed for the successful and safe taper of prednisone to a minimal dose, thereby mitigating the long-term risks associated with GCs therapy. The continued remission on monthly tocilizumab monotherapy underscores the importance of maintaining targeted immunosuppression to prevent relapse of both articular and extra-articular disease.

An important consideration is whether the improvement in neuropathy was a direct effect of tocilizumab on nerve vasculature or secondary to the control of the systemic RA flare. Given the shared pathophysiological driver—IL-6-mediated systemic inflammation and endothelial injury—it is most plausible that tocilizumab simultaneously addressed both phenomena. The striking temporal correlation between IL-6 receptor blockade, normalization of inflammatory markers, and the objective electrophysiological recovery supports the conclusion that suppressing this central cytokine pathway resolved the common inflammatory substrate responsible for both the articular and neuropathic manifestations.

## Conclusion

This case underscores that severe, refractory PN in RA is a serious complication often linked to uncontrolled systemic inflammation and vasculitis. While high-dose GCs serve as a critical rescue therapy, their substantial toxicity profile necessitates a swift transition to a steroid-sparing, targeted biologic agent. Tocilizumab presents a compelling therapeutic choice in this context, given the central role of IL-6 in RA pathogenesis, its proven efficacy in refractory arthritis, emerging evidence in systemic manifestations, and its ability to facilitate rapid GCs taper. The presented therapeutic sequence—acute control with GCs followed by rapid induction of IL-6 inhibition—proved highly effective, leading to the resolution of inflammatory activity, significant neurological recovery, and minimization of long-term treatment-related risks. This case contributes to the accumulating evidence supporting the utility of tocilizumab in the management of complex, extra-articular RA and highlights the need for a treat-to-target approach that addresses both joint and systemic disease.

## Data Availability

The original contributions presented in the study are included in the article/supplementary material. Further inquiries can be directed to the corresponding author.

## References

[1] Zhang Y Yang W Xiong W Chen Y Zhuang P Wang H . Disrupting complement-inflammation positive feedback circuit via oligonucleotide hydrogel microspheres for reversing joint inflammation. Adv Mater. (2025), e18378. doi: 10.1002/adma.202518378, PMID: 41312643

[2] Zhang Y Liu D Chen W Tao Y Li W Qi J . Microenvironment-activatable probe for precise NIR-II monitoring and synergistic immunotherapy in rheumatoid arthritis. Adv Mater. (2024) 36:e2409661. doi: 10.1002/adma.202409661, PMID: 39370578

[3] Jin W Wang Q Jin C Xue M Pan L Zeng Y . Spatiotemporal distributions and regional disparities of rheumatoid arthritis in 953 global to local locations, 1980-2040, with deep learning-empowered forecasts and evaluation of interventional policies’ benefits. Ann Rheum Dis. (2025) 84:1104–16. doi: 10.1016/j.ard.2025.04.009, PMID: 40527715

[4] Bhattacharyya S Helfgott SM . Neurologic complications of systemic lupus erythematosus, sjögren syndrome, and rheumatoid arthritis. Semin Neurol. (2014) 34:425–36. doi: 10.1055/s-0034-1390391, PMID: 25369438

[5] de Araújo Pereira F de Almeida Lourenço M de Assis MR . Evaluation of peripheral neuropathy in lower limbs of patients with rheumatoid arthritis and its relation to fall risk. Adv Rheumatol. (2022) 62:9. doi: 10.1186/s42358-022-00238-3, PMID: 35317839 PMC8938971

[6] Abdelrazek MS Elnaggar BMA Ahmed AM Mohamed MSE . Clinical, neurophysiological, and radiological characteristics of peripheral neuropathy in patients with rheumatoid arthritis: A cross-sectional observational study. J Neurol Sci. (2025) 473:123523. doi: 10.1016/j.jns.2025.123523, PMID: 40359775

[7] Albani G Ravaglia S Cavagna L Caporali R Montecucco C Mauro A . Clinical and electrophysiological evaluation of peripheral neuropathy in rheumatoid arthritis. J Peripher Nerv Syst. (2006) 11:174–5. doi: 10.1111/j.1085-9489.2006.00084.x, PMID: 16787518

[8] Puéchal X Said G Hilliquin P Coste J Job-Deslandre C Lacroix C . Peripheral neuropathy with necrotizing vasculitis in rheumatoid arthritis. A clinicopathologic and prognostic study of thirty-two patients. Arthritis Rheum. (1995) 38:1618–29. doi: 10.1002/art.1780381114, PMID: 7488283

[9] Tanemoto M Hisahara S Hirose B Ikeda K Matsushita T Suzuki S . Severe mononeuritis multiplex due to rheumatoid vasculitis in rheumatoid arthritis in sustained clinical remission for decades. Intern Med. (2020) 59:705–10. doi: 10.2169/internalmedicine.3866-19, PMID: 31735796 PMC7086314

[10] Makol A Crowson CS Wetter DA Sokumbi O Matteson EL Warrington KJ . Vasculitis associated with rheumatoid arthritis: a case-control study. Rheumatol (Oxford). (2014) 53:890–9. doi: 10.1093/rheumatology/ket475, PMID: 24441152 PMC3999374

[11] Mertz P Wollenschlaeger C Chasset F Dima A Arnaud L . Rheumatoid vasculitis in 2023: Changes and challenges since the biologics era. Autoimmun Rev. (2023) 22:103391. doi: 10.1016/j.autrev.2023.103391, PMID: 37468085

[12] Duru N van der Goes MC Jacobs JW Andrews T Boers M Buttgereit F . EULAR evidence-based and consensus-based recommendations on the management of medium to high-dose glucocorticoid therapy in rheumatic diseases. Ann Rheum Dis. (2013) 72:1905–13. doi: 10.1136/annrheumdis-2013-203249, PMID: 23873876

[13] Bergstra SA Sepriano A Kerschbaumer A van der Heijde D Caporali R Edwards CJ . Efficacy, duration of use and safety of glucocorticoids: a systematic literature review informing the 2022 update of the EULAR recommendations for the management of rheumatoid arthritis. Ann Rheum Dis. (2023) 82:81–94. doi: 10.1136/ard-2022-223358, PMID: 36410794

[14] Tektonidou MG Serelis J Skopouli FN . Peripheral neuropathy in two patients with rheumatoid arthritis receiving infliximab treatment. Clin Rheumatol. (2007) 26:258–60. doi: 10.1007/s10067-006-0317-z, PMID: 16683176

[15] Garcia-Porrua C González-Gay MA . Successful treatment of refractory mononeuritis multiplex secondary to rheumatoid arthritis with the anti-tumour necrosis factor alpha monoclonal antibody infliximab. Rheumatol (Oxford). (2002) 41:234–5. doi: 10.1093/rheumatology/41.2.234, PMID: 11886980

[16] Kunchok A Aksamit AJ Jr. Davis JM 3rd Kantarci OH Keegan BM Pittock SJ . Association between tumor necrosis factor inhibitor exposure and inflammatory central nervous system events. JAMA Neurol. (2020) 77:937–46. doi: 10.1001/jamaneurol.2020.1162, PMID: 32421186 PMC7235930

[17] Plushner SL . Tocilizumab: an interleukin-6 receptor inhibitor for the treatment of rheumatoid arthritis. Ann Pharmacother. (2008) 42:1660–8. doi: 10.1345/aph.1L268, PMID: 18957621

[18] Iijima T Suwabe T Sumida K Hayami N Hiramatsu R Hasegawa E . Tocilizumab improves systemic rheumatoid vasculitis with necrotizing crescentic glomerulonephritis. Mod Rheumatol. (2015) 25:138–42. doi: 10.3109/14397595.2013.874748, PMID: 24533557

[19] DeQuattro K Imboden JB . Neurologic manifestations of rheumatoid arthritis. Rheum Dis Clin North Am. (2017) 43:561–71. doi: 10.1016/j.rdc.2017.06.005, PMID: 29061242

[20] Syngle A Verma I Krishan P . Interleukin-6 blockade improves autonomic dysfunction in rheumatoid arthritis. Acta Reumatol Port. (2015) 40:85–8., PMID: 24863079

[21] Abdulqader Y Al-Ani M Parperis K . Rheumatoid vasculitis: early presentation of rheumatoid arthritis. BMJ Case Rep. (2016). doi: 10.1136/bcr-2016-217557, PMID: 27873751 PMC5129099

[22] Navarro EP Posso-Osorio I Aguirre-Valencia D Naranjo-Escobar J Tobón GJ . Tofacitinib and risk of peripheral neuropathy? Experience of 2 cases in patients with rheumatoid arthritis. J Clin Rheumatol. (2021) 27:e58–60. doi: 10.1097/rhu.0000000000000864, PMID: 30028803

[23] Richards BL Spies J McGill N Richards GW Vaile J Bleasel JF . Effect of leflunomide on the peripheral nerves in rheumatoid arthritis. Intern Med J. (2007) 37:101–7. doi: 10.1111/j.1445-5994.2007.01266.x, PMID: 17229252

[24] Martin-Iglesias D Paredes-Ruiz D Ruiz-Irastorza G . Use of glucocorticoids in SLE: A clinical approach. Mediterr J Rheumatol. (2024) 35:342–53. doi: 10.31138/mjr.230124.uos, PMID: 39193186 PMC11345604

[25] Oldfield V Dhillon S Plosker GL . Tocilizumab: a review of its use in the management of rheumatoid arthritis. Drugs. (2009) 69:609–32. doi: 10.2165/00003495-200969050-00007, PMID: 19368420

[26] Jarlborg M Gabay C . Systemic effects of IL-6 blockade in rheumatoid arthritis beyond the joints. Cytokine. (2022) 149:155742. doi: 10.1016/j.cyto.2021.155742, PMID: 34688020

[27] Oldenburg HS Rogy MA Lazarus DD Van Zee KJ Keeler BP Chizzonite RA . Cachexia and the acute-phase protein response in inflammation are regulated by interleukin-6. Eur J Immunol. (1993) 23:1889–94. doi: 10.1002/eji.1830230824, PMID: 8344351

[28] Boss B Neeck G . Correlation of IL-6 with the classical humoral disease activity parameters ESR and CRP and with serum cortisol, reflecting the activity of the HPA axis in active rheumatoid arthritis. Z Rheumatol. (2000) 59 Suppl 2:62–4. doi: 10.1007/s003930070020, PMID: 11155806

[29] López-Mejías R González-Gay MA . IL-6: linking chronic inflammation and vascular calcification. Nat Rev Rheumatol. (2019) 15:457–9. doi: 10.1038/s41584-019-0259-x, PMID: 31235835

[30] Rojas M Zhang W Lee DL Romero MJ Nguyen DT Al-Shabrawey M . Role of IL-6 in angiotensin II-induced retinal vascular inflammation. Invest Ophthalmol Vis Sci. (2010) 51:1709–18. doi: 10.1167/iovs.09-3375, PMID: 19834028 PMC2868419

[31] Davies R Williams J Sime K Jin HS Thompson C Jordan L . The role of interleukin-6 trans-signalling on cardiovascular dysfunction in inflammatory arthritis. Rheumatol (Oxford). (2021) 60:2852–61. doi: 10.1093/rheumatology/keaa725, PMID: 33313793 PMC8213430

[32] Taylor PC Feist E Pope JE Nash P Sibilia J Caporali R . What have we learnt from the inhibition of IL-6 in RA and what are the clinical opportunities for patient outcomes? Ther Adv Musculoskelet Dis. (2024) 16:1-19. doi: 10.1177/1759720x241283340, PMID: 39444594 PMC11497505

[33] Oba Y Sawa N Ikuma D Mizuno H Inoue N Sekine A . Successful peficitinib monotherapy for the new-onset skin manifestations of rheumatoid vasculitis after long-term treatment with tocilizumab for rheumatoid arthritis. Mod Rheumatol Case Rep. (2023) 8:5–10. doi: 10.1093/mrcr/rxad025, PMID: 37210210

[34] Emery P Keystone E Tony HP Cantagrel A van Vollenhoven R Sanchez A . IL-6 receptor inhibition with tocilizumab improves treatment outcomes in patients with rheumatoid arthritis refractory to anti-tumour necrosis factor biologicals: results from a 24-week multicentre randomised placebo-controlled trial. Ann Rheum Dis. (2008) 67:1516–23. doi: 10.1136/ard.2008.092932, PMID: 18625622 PMC3811149

[35] Puéchal X Gottenberg JE Berthelot JM Gossec L Meyer O Morel J . Rituximab therapy for systemic vasculitis associated with rheumatoid arthritis: Results from the AutoImmunity and Rituximab Registry. Arthritis Care Res (Hoboken). (2012) 64:331–9. doi: 10.1002/acr.20689, PMID: 22076726

